# Heat Transport Compensation in Atmosphere and Ocean over the Past 22,000 Years

**DOI:** 10.1038/srep16661

**Published:** 2015-11-16

**Authors:** Haijun Yang, Yingying Zhao, Zhengyu Liu, Qing Li, Feng He, Qiong Zhang

**Affiliations:** 1 Laboratory for Climate and Ocean-Atmosphere Studies (LaCOAS) and Department of Atmospheric and Oceanic Sciences, School of Physics, Peking University, Beijing 100871, China; 2 Qingdao Collaborative Innovation Center of Marine Science and Technology, Qingdao 266003, China; 3 Center for Climatic Research, University of Wisconsin, Madison, Wisconsin 53706, USA; 4 Department of Earth, Environmental and Planetary Sciences, Brown University, Rhode Island 02912, USA; 5 Department of Physical Geography and Bolin Centre for Climate Research, Stockholm University, Stockholm 10691, Sweden

## Abstract

The Earth’s climate has experienced dramatic changes over the past 22,000 years; however, the total meridional heat transport (MHT) of the climate system remains stable. A 22,000-year-long simulation using an ocean-atmosphere coupled model shows that the changes in atmosphere and ocean MHT are significant but tend to be out of phase in most regions, mitigating the total MHT change, which helps to maintain the stability of the Earth’s overall climate. A simple conceptual model is used to understand the compensation mechanism. The simple model can reproduce qualitatively the evolution and compensation features of the MHT over the past 22,000 years. We find that the global energy conservation requires the compensation changes in the atmosphere and ocean heat transports. The degree of compensation is mainly determined by the local climate feedback between surface temperature and net radiation flux at the top of the atmosphere. This study suggests that an internal mechanism may exist in the climate system, which might have played a role in constraining the global climate change over the past 22,000 years.

The Earth’s climate has experienced remarkable changes since the Last Glacial Maximum^1^,^2^,^3^ (LGM, 21 ka; ka = thousand years ago). The surface climate in the Northern Hemisphere (NH) experienced significant cooling during the Older Dryas (OD, 19.0–14.5 ka; also known as the Heinrich Event 1, or H1, ~17 ka). Because of retreat of the continental glacier and massive melting of sea ice, the Atlantic meridional overturning circulation (AMOC) almost shut down^2^,^3^,^4^,^5^ and the North Atlantic sea surface temperature (SST) dropped by more than 4 °C during the OD. The H1 was then followed by an abrupt warming occurred at the onset of the Bølling-Allerød^6^,^7^,^8^,^9^,^10^,^11^,^12^ (BA, ~14.5 ka). The atmospheric circulation over the North Atlantic region flipped within just a few years to another state^13^ and the Greenland temperatures skyrocketed by more than 10 °C over only a few decades^8^, terminating the H1 cold phase, accompanied by a rapid recovery and even overshooting of the AMOC above the LGM level^3^. Since then, the Earth’s climate experienced again a short period of Younger Dryas cold phase (YD, ~12.8 ka) and subsequently a slow warming since the start of the Holocene^14^ (11.5 ka–present).

The Earth’s energy balance is maintained by hemispherically antisymmetric meridional heat transports (MHTs). A long transient simulation (CCSM3 TraCE-21 K) (Methods) on the climate from the LGM to the pre-industrial^3^,^15^ is used to study the evolution of the MHTs during the past 22 kyr (1 kyr = 1000 years). This long simulation captured many major features of the deglacial climate evolution^3^,^14^,^15^,^16^,^17^, including the magnitude of the climate response as inferred from paleo-proxy archives, by prescribing the temporal evolution of the external boundary conditions based on astronomical theory, ice-sheet reconstruction and the history of greenhouse gas concentration. This simulation also showed that the global total MHT, however, has been undulating in a narrow range throughout the past 22 kyr despite of those remarkable changes in Earth’s temperature and ocean circulations, such like the “on” and “off” states of the AMOC as well as the CO_2_ concentration change. We identified that the small fluctuation in global overall MHT results from compensating changes in the two components of the MHT: the atmosphere heat transport (AHT) and the ocean heat transport (OHT). A coupled box model was used to understand the mechanism at work^18^ (Supplementary). This box model was able to reproduce conceptually the evolutions of AMOC and MHT since the LGM. It showed that the Earth’s energy conservation forces the compensation changes between AHT and OHT; it further revealed that the degree of compensation is determined by internal parameters of the climate system.

## Results

### Meridional heat transport

**Figure 1:**
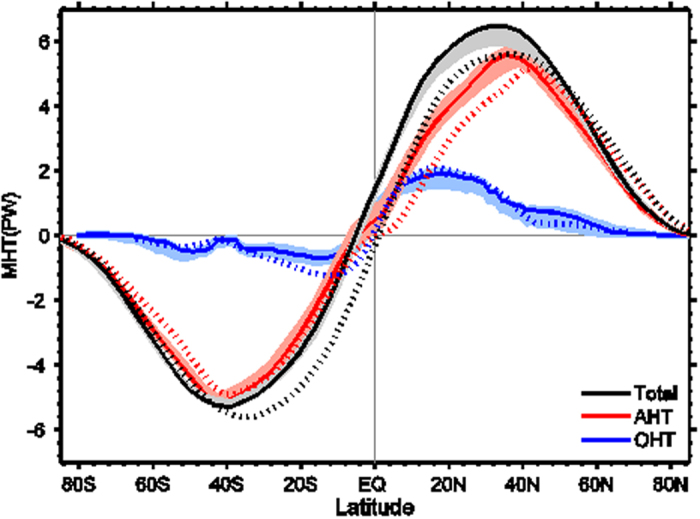
Meridional heat transport (MHT). Black curve is the combined heat transport by the atmosphere and ocean; red, atmosphere heat transport (AHT); and blue, ocean heat transport (OHT). Units: PW; 1 PW = 10^15^ W. Each solid curve represents the mean heat transport during the LGM period (22–20 ka). Light color curve shows the spread of corresponding heat transport since the LGM. The data source is the CCSM3 TraCE-21 K simulation. Dotted curves are the corresponding heat transports based on present-day observations^19^.

Despite of significant change of the global climate since the LGM, the global MHT deviated little from the long-term mean (Fig. 1). The Earth’s climate is maintained by a hemispherically antisymmetric poleward heat transport with the peak value of about 5.5 PW (1 PW = 10^15^ W) at 35–40°N/S. The AHT dominates in the region poleward of about 30°N/S, while the OHT dominates in the deep tropics^19^,^20^,^21^,^22^. These features were nearly constant throughout the past 22 kyr (Fig. 1). The maximum deviation of total MHT was less than 0.4 PW, or within 7% of the long-term mean, much lower than the percentage changes occurred in the AMOC and the global temperature during the H1 event when the AMOC was weakened by nearly 70% and the tropical SST over the Atlantic was cooled significantly^3^. The past MHTs were also close to the present-day MHTs (dotted curves in Fig. 1) based on the present observations^19^. The mean MHTs over the LGM period lay in the upper limits of their ranges (solid curves in Fig. 1), with deviations within 10% of the present-day observation. It is striking to see that the OHT throughout the past 22 kyr was in an excellent agreement with the present-day OHT, regardless of the “on” and “off” states of the AMOC. It is well recognized that the AMOC plays a significant role in the northward heat transport and that its collapse would substantially reduce the northward OHT^23^. The stable OHT implies that the heat transport reduction due to the AMOC should be compensated by the heat transport increase in the Pacific, suggesting a so-called interbasin seesaw relationship between the Indo-Pacific and Atlantic in both mass transport and energy transport^24^,^25^,^26^. This interbasin relation will be investigated in detail in our future paper. Here, we focus on the relationship between the changes in AHT and OHT.

### Compensating changes

**Figure 2:**
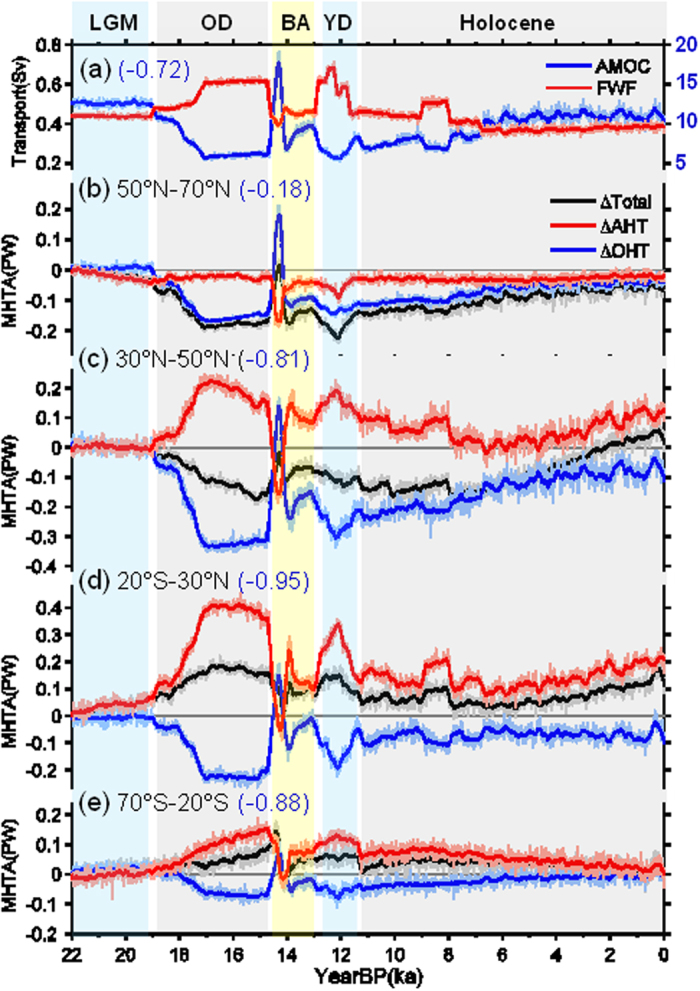
AMOC, FWF and MHT. (**a**) The blue curve is the AMOC index, defined as the maximum value of the streamfunction over 20°–70°N and between 300–2000 m in the Atlantic (Units: Sv; 1 Sv = 10^6^ m^3^s^−1^); red curve is the FWF (Sv), obtained by integrating the total meltwater flux over the North Atlantic between 35° and 70°N. The number in parentheses indicates the correlation coefficient between the AMOC and FWF. (**b–e**) Anomalous MHT averaged over different latitude bands (PW). The linear trends of heat transports over the whole 22 kyr are removed first, and then the mean values at 22 ka are subtracted. In (**b–e**) the black curve is the total MHT; red, the AHT; and blue, the OHT. The number in parentheses indicates correlation coefficient between AHT and OHT. The thick curves in (**a–e)** are the low-pass-filtered (150-year running mean) versions of the corresponding time series.

The stable total MHT results from the compensating changes in AHT and OHT, which is particularly clear during the OD (Fig. 2). The transient changes in MHTs, relative to the mean LGM state in 22 ka, are examined, together with variations in the AMOC and freshwater forcing in the North Atlantic (Methods). The changes in AHT and OHT compensated each other at most latitudes from the LGM to the OD; since then, the AHT and OHT remained negatively correlated though the compensation between them seems to fail (Fig. 2c–e). Figure 2 also shows that there is no compensation in the Northern Hemisphere between 50°–70°N (Fig. 2b). At these latitudes, the mean OHT can be neglected (Fig. 1) and the mean AHT is small when compared to that in the lower latitudes. Since the MHT in high latitudes plays a minor role in global energy balance, we will focus on compensation between AHT and OHT in lower latitudes in this paper.

**Figure 3:**
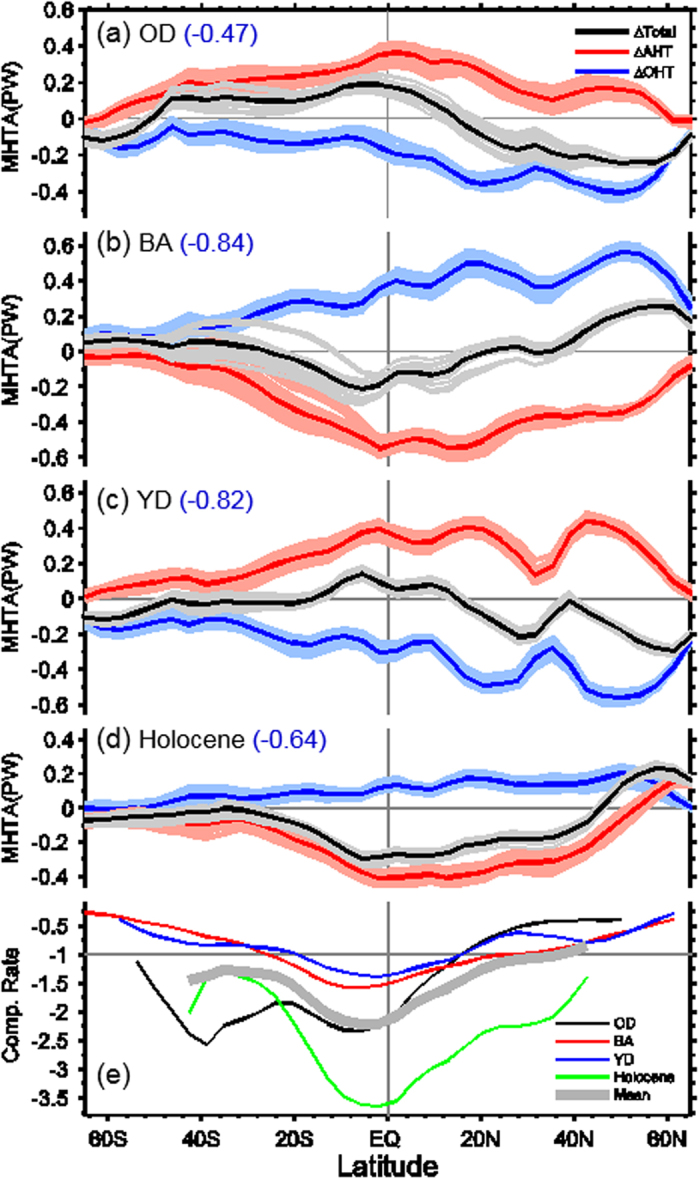
MHT and BJC rate. (**a–d**) The black curve is the total anomalous MHT; red, the anomalous AHT; and blue, the anomalous OHT. Units: PW. Anomalous heat transports are obtained by simply subtracting the mean values in the immediately preceding period (see Methods). Thick solid curves represent temporally averaged anomalous heat transports, and light color curves show the spread of anomalous heat transports. The number in parentheses indicates correlation coefficient between AHT and OHT. (**e**) BJC rate, defined as the ratio of anomalous AHT to anomalous OHT. The black, red, blue, and green curves are for the BJC rates during the OD, BA, YD, and Holocene, respectively. The thin grey line of -1 represents perfect compensation. The thick grey curve in (**e**) is the mean BJC rate for all periods.

The compensating changes in AHT and OHT occurred actually over the whole 22 kyr and at most latitudes, following these significant climate shifts: from the LGM to the OD, from the OD to the BA, from the BA to the YD, and even in the mild climate evolution period during the Holocene (Fig. 3), if we compare the changes in AHT and OHT relative to the mean state in the immediate preceding period. This is the so-called “Bjerknes compensation” (BJC), first proposed by Bjerknes in 1964^27^. Under the constraint of global energy conservation, the steady climate requires that any large variations in ocean and atmosphere energy transports should be equal in magnitude but have opposite signs^27^,^28^. This negative relationship is a fundamental feature of the climate system^28^. However, the BJC does not rule out large changes in both ocean and atmosphere^28^,^29^ (Fig. 2a), nor in regional climate^29^. The BJC could be a critical constraint to the climate system, and may play a role in the stability of overall Earth’s climate. It may also suggest a potential self-restoring mechanism in a complex climate system.

Figure 3 also shows that the compensation changes in AHT and OHT can exhibit different features. The AHT change can either overcompensate or undercompensate the OHT change, varying with latitude and period. The BJC rate, defined as the ratio of AHT change to OHT change (Fig. 3e), is about −1.5 (thick grey curve in Fig. 3e), which suggests that in general the AHT change overcompensates the OHT change by about 50%. This rate differs slightly for different periods. The best compensation occurred during the BA and the YD (Fig. 3b,c), with the overcompensation (undercompensation) in the range of 20% (Fig. 3e). The worst compensation occurred during the Holocene, with the BJC rate of about −2.0 to −3.0, suggesting the AHT change being 2–3 times of the OHT change (Fig. 3d, green curve in Fig. 3e), when the YD mean state is used. We can also see that, in general, the AHT change overcompensates the OHT change in the tropics (20°S–20°N) and undercompensates it in the extratropics.

A fundamental question is why the BJC occurred over the past 22 kyr. The BJC has been shown to be valid in the internal climate variability^30^,^31^,^32^, or under significant external forcing^23^,^33^,^34^,^35^,^36^,^37^,^38^,^39^,^40^. However, for a coupled ocean-atmosphere system in a quasi-equilibrium state (so that the change in oceanic heat storage is negligible), the total MHT is determined by the energy flux at the top of the atmosphere (TOA), which in turn is determined by climate feedbacks in the coupled climate system^21^,^28^,^30^,^32^,^34^,^37^, such as the sea-ice-albedo positive feedback in the polar region, the negative feedback between outgoing longwave flux and surface temperature. It is not obvious why the BJC should be valid over the past 22 kyr, because during that period the Earth climate had experienced significant changes; for instance, strong climate feedback had caused the shutdown of the AMOC in the OD. The latter can also result in significant change in the TOA energy flux and thus the total MHT^34^.

### Simulations in a conceptual model

A conceptual coupled box model has been proposed to investigate the compensating changes in AHT and OHT^18^. The model consists of a 2-box atmosphere and a 4-box ocean (Methods and Supplementary). The simple model study has shown that, under reasonable perturbation, the BJC is valid in the presence of climate feedback. Actually, the climate feedback is critical to the BJC’s behaviours and eventually determines the degree of compensation. The BJC was always valid in the past 22 kyr due to the constraint of global energy conservation, which is implied by the quasi-equilibrium total MHT (Fig. 1).

The analytical solution to the BJC is obtained under the constraint of global energy conservation (Methods). The BJC rate 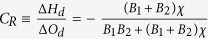, which actually depends only on two internal parameters of the coupled climate system: the local climate feedback parameter 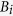 (the subscripts i = 1, 2 refer to the extratropical and tropical boxes, respectively) between surface temperature and the TOA energy flux, and the atmosphere heat transport efficiency 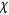
29,41. Previous studies noted that 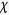 is always positive and less uncertain^28^,^42^,^43^. 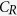 is thus mainly determined by climate feedback parameter 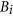. 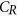 was also studied in a two-box energy balance model^44^ without considering ocean dynamics. In a stable climate system, 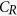 is always negative, which means in principle the compensation is always valid. However the degree of compensation can have three different features, depending on 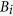. The AHT can perfectly compensate the OHT (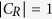) if there is no climate feedback anywhere (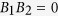), or undercompensate the OHT (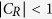) if there is negative feedback everywhere (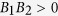), or overcompensate the OHT (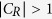) if there are opposite feedbacks in tropics and extratropics (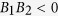).

The key climate parameters used in the box model can be diagnosed from the transient 22-kyr CCSM3 simulation (Supplementary). The AHT coefficient 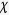 is about 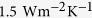. During the past 22 kyr, the averaged climate feedback parameters for the extratropical 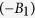 and tropical 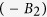 boxes were 0.4 and 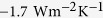, respectively, representing a weak positive feedback in the extratropics and a strong negative feedback in the tropics. The analytical solution 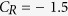, suggesting that the AHT will overcompensate the OHT by about 50%, being well consistent with the compensation rate shown in Fig. 3e.

**Figure 4:**
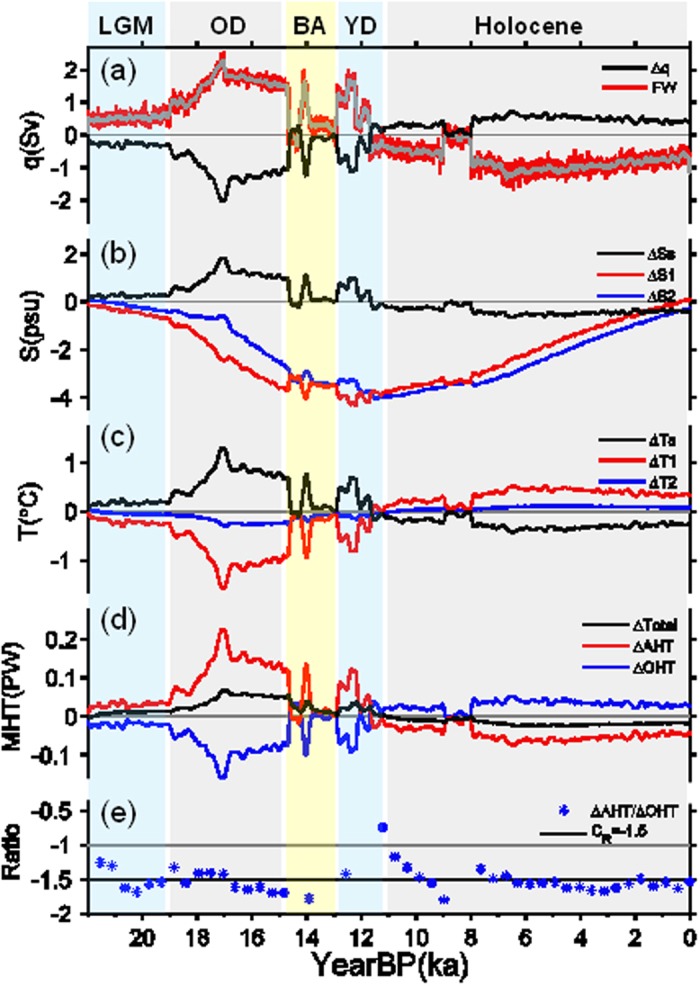
Box model simulations in response to FWF over the past 22 kyr. (**a**) The black curve is mass transport change (Sv); red and grey, the FWF (0.1 Sv), similar to those in Fig. 2a but with reduced amplitude. (**b)** Salinity changes (psu) in surface extratropical box (red) and surface tropical box (blue); the black curve is the change in meridional sea surface salinity (SSS) gradient. (**c**) Same as (**b**) except for temperature changes (°C). (**d**) Changes in total MHT (black), AHT (red) and OHT (blue) in PW. (**e**) BJC rate. The black line is the analytical value (−1.5) based on Eq. (18) (see Methods), and blue asterisks indicate the transient BJC rate from the coupled box model.

Conceptually, the climate evolution over the past 22 kyr is reasonably reproduced by the box model (Fig. 4). Forced by freshwater flux (FWF) in the extratropics (Methods), the variation of the northward mass transport in the box model (which mimics the AMOC in the real world) (Fig. 4a) is in a good agreement with the AMOC in the CCSM3 (Fig. 2a), except for a weaker magnitude. The H1, BA and YD events are all well simulated in the box model, in responses to the enhanced FWF during 19–15 ka, the suddenly weakened FWF around 14.5 ka and the re-enhanced FWF around 12.8 ka, respectively (Fig. 4a). The ocean component of the box model was generally freshening due to the FWF input until 12 ka, and then reversed the freshening trend toward the present climate due to the FWF reduction (Fig. 4b). The poleward sea surface salinity (SSS) gradient (Fig. 4b) followed closely the variation of FWF, intensifying during 22–19 ka and weakening gradually since 12 ka. The ocean component of the box model became colder until 12 ka and then warmed up gradually toward the present temperature (Fig. 4c), which, however, mainly occurred in the extratropical ocean (red curve in Fig. 4c), while the tropical SST hardly changed (blue curve in Fig. 4c). The change in poleward SST gradient was in phase with that in poleward SSS gradient; both of them were out of phase with mass transport (Fig. 4a). One can see that the salinity change (Fig. 4b) dominated the mass transport change (Fig. 4a), which in turn altered the ocean temperature (Fig. 4c).

The box model exhibits clearly the BJC (Fig. 4d). Taking the H1 event during the OD as an example, the FWF input freshened the surface ocean in high latitudes, resulting in a weakening of the mass transport, which reduced the poleward OHT (Fig. 4d) and thus the high-latitude temperature (Fig. 4c), increasing the poleward SST gradient. The enhanced SST gradient increased the atmospheric baroclinicity, resulting in an increase in AHT (Fig. 4d), which compensated the reduction in OHT. The transient BJC rate is shown in Fig. 4e, which oscillates closely around the analytical value (−1.5). Note that ocean dynamics is deeply involved in this process^18^. It is the salinity change that controlled the variation of thermohaline circulation. The climate changes over other periods followed a similar rationale.

### Mechanism

**Figure 5:**
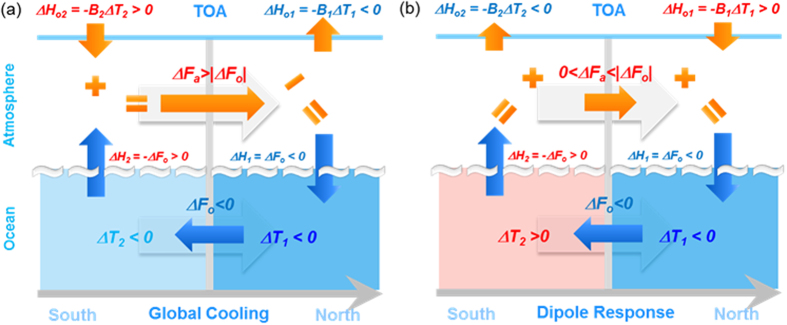
BJC mechanism. (**a**) Under a weak positive feedback in the extratropical box (–*B*_*1*_ = 0.4, *B*_*1*_*B*_*2*_ < 0), anomalous AHT has to overcompensate anomalous OHT in order to keep the energy conserved in both tropical and extratropical boxes. (**b**) Under negative feedbacks in both the extratropical and tropical boxes (*B*_*1*_*B*_*2*_ > 0), the anomalous AHT undercompensates the anomalous OHT. Two wide grey background arrows denote the mean heat transports. See Methods for the meanings of the symbols.

Now, we can neatly address the BJC mechanism (Fig. 5). Again, taking the climate change during the H1 event as an example, the decreased poleward OHT led to an extratropical cooling (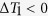; Fig. 4c). Due to the local positive feedback (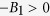) (for example, positive shortwave cloud radiative feedback associated with sea-ice change), the extratropical cooling increased the outgoing TOA energy flux (Fig. 5a). This further enhanced the extratropical cooling. Therefore, to maintain the energy balance, the extratropical box needed heat import from the tropics through the atmosphere to offset both the outgoing TOA energy flux and the decreased OHT. The anomalous poleward AHT had to be more than (overcompensate) the anomalous OHT (Fig. 5a). For the tropical box, the excessive heat export to the extratropical box through the atmosphere led to a weaker cooling (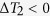). This tropical cooling increased the TOA energy gain because of strong negative feedback (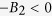), and therefore could sustain the extra heat loss in the extratropical region. In this case, the decreased poleward OHT led to a cooling everywhere and resulted in a global mean cooling (Fig. 5a).

In the case of negative climate feedback everywhere (Fig. 5b), the local cooling tended to reduce the heat loss into the space, so that the compensating AHT would always be weaker than the perturbation OHT, leading to undercompensation. In any case, it is the global energy conservation that requires opposite changes in AHT and OHT. In other words, the compensating changes in AHT and OHT helped to maintain the equilibrium climate. Of course, the compensation is not perfect unless there is no feedback between the TOA flux and surface temperature (i.e., 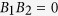). Quantitatively, as the negative feedback intensifies (larger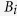), the energy balance tends to be fulfilled locally^18^,^38^, instead of by horizontal redistribution; therefore, the BJC will become less significant (Supplementary).

## Discussion

The simple box model captured some fundamental features of the climate system despite of its simplicity. It suggests that the BJC may have played an important role in constraining dramatic shift of the overall Earth’s climate over the past 22 kyr, even in the presence of strong feedback between the TOA energy flux and surface temperature. The BJC may have acted as an invisible hand trying to maintain the energy balance of the Earth climate, although the Earth has been gradually warming up since the LGM^14^.

The accumulated heat imbalance due to CO_2_ change since the LGM is about 3.3 W/m^2^. This corresponds to a yearly heat perturbation of about 1.5 × 10^−4^ W/m^2^. Over such a long period, its effect on the MHT is negligible when compared with the freshwater effect. Our results reported in another paper^18^ showed that the FWF played a dominant role in the AMOC change over the past 22 kyr, with respect to the warming effect of CO_2_. Therefore, the BJC was always valid in the past 22 kyr. However, the accumulated CO_2_-related heat imbalance since the pre-industrial is around 1.66 ± 0.17 W/m^2^, or around 1 × 10^−2^ W/m^2^ yearly^45^. Whether the BJC is valid during this short period needs to be investigated thoroughly. In addition, whether this mechanism is working or not in the present climate change, and in future climate change, under rapid increase of CO_2_, is a serious concern to us.

We are fully aware of the shortcomings of the coupled box model. The box model is constructed based on a Stommel-like hemispheric box model and includes only a buoyancy-driven overturning circulation. It does not include the wind-driven circulation. Heat transport by wind-driven circulations in the Indo-Pacific also has important contribution to the global energy balance, particularly in the tropics. The consistency of CCSM Trace-21 K and the coupled box model suggests that qualitatively the compensation feature would not change without wind-driven circulation. Our box model considers an equator-to-pole scale overturning circulation and does not include the Southern Ocean. It should be tested for the inter-hemispheric exchange across the equator. The parameterization of AHT in the box model is too simple, without the contribution of the Hadley Cell. In the CCSM, the Hadley Cell is strongly coupled with the wind-driven circulation, which changes the degree of compensation to some extent. Nevertheless, the simple coupled box model can help us understand some fundamental mechanisms of heat transport changes over the past 22 kyr. Comprehensive studies are needed to test whether or not the BJC is truly robust in the real Earth climate.

## Methods

### Coupled climate model and simulation

The coupled general circulation model is the Community Climate System Model version 3 (CCSM3)^46^ maintained at the National Center for Atmospheric Research (NCAR), with the resolution of T31_gx3v5. A Transient simulation of Climate Evolution of the last 21,000 years (TraCE-21 K) is used here^3^,^15^,^17^. This experiment was initialized using output from an equilibrium simulation forced by the LGM forcing (22 ka), and then forced by a complete set of realistic transient climate forcing, orbital insolation^47^, atmospheric greenhouse gases^48^, and meltwater discharge. The coastlines and bathymetry were modified at 13.1 ka with the removal of the Fennoscandian Ice Sheet from the Barents Sea, at 12.9 ka with the opening of the Bering Strait, at 7.6 ka with the opening of Hudson Bay, and finally at 6.2 ka with the opening of the Indonesian Throughflow^15^. Meltwater flux is mainly based on the records of sea level rise and geological indicators of ice sheet retreat and meltwater discharge. The meltwater forcing during mwp-1 A consists of contributions from the Antarctic (15 m of equivalent sea-level volume) and Laurentide (5 m of equivalent sea-level volume) Ice Sheets. Details of the TraCE-21 K can be found in ref. 15. The TraCE-21 K reproduced many key features of the response of the climatology in the last 22 kyr as in the reconstructions^3^,^14^,^17^, such as the AMOC intensity^3^,^17^,^49^, cross-equator SST contrast^3^,^17^ and tropical Pacific SST^3^,^17^.

The AHT and OHT are directly calculated from the model velocity and temperature^50^. This ensures the independent calculation of AHT and OHT. The total MHT is the sum of AHT and OHT. To obtain Fig. 2, the linear trends of the heat transports over the past 22 kyr are removed first, and then the mean values during the 22 kyr are subtracted in order to examine the heat transport changes since the LGM. For Fig. 3 that is to show the relative changes of the heat transports during different periods, the linear trends are not removed and the heat transport changes are obtained by simply subtracting the mean state in the immediately preceding period from the heat transports in the targeted period. The BJC rate is defined as the ratio of anomalous AHT to anomalous OHT.

### Conceptual coupled box model and simulation

The box model consists of a 2-box atmosphere and a 4-box ocean^18^ (Supplementary). The 2-box atmosphere covers one hemisphere and the 4-box ocean spans an arbitrary longitude sector (60° for the Atlantic sector or 180° for the global NH ocean) between the equator and 75°N. The atmosphere is assumed to mix the heat perfectly in the zonal direction, so that the oceanic influence on the atmosphere energy budget is zonally homogeneous. All the boxes are connected at 35°N, where the zonal mean net radiative forcing is close to zero and the northward transports of heat and moisture in the atmosphere are near their peaks. The zonal mean net radiative forcing is positive (negative) to the south (north) of 35°N. The 2-box ocean model is based originally on Stommel (1961)^51^ and developed further in many studies^29^,^41^,^52^,^53^. More details can be found in Supplementary.

The ocean boxes are governed by eight equations:


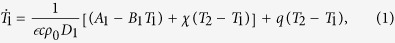



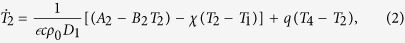



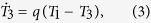



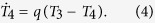



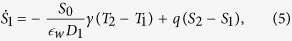



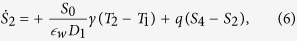



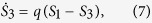



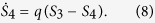


where 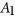 (<0) and 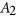 (>0) are net incoming radiation (Wm^−2^) at high and low latitudes, respectively; 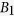 and 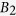 are climate feedback parameters at high and low latitudes, respectively; 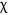 and 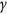 are the efficiencies of atmosphere heat and moisture transports, respectively; 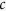 is the seawater specific heat capacity; 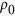 is the seawater density; 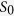 is a constant reference salinity (35 psu). 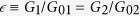, which indicates relative ocean coverage in both high and low latitude areas; here, 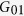 and 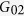 are the entire areas of the two atmosphere boxes separated by 35°N; 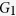 and 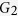 are the areas of corresponding ocean boxes. For simplicity, we assume 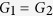 and 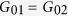. If an aquaplanet is studied, 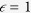; otherwise, 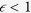. 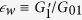, where 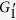 depicts the ocean area as well as the catchment area of the ocean basin, which includes the influence of river runoff on the oceanic freshwater budget. It is obvious that 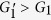. All parameters used in this study can be found in Table 1 of Supplementary.

The coupled box model is driven by energy flux at the TOA. The net radiative forcing (Wm^−2^) at the TOA can be linearly parameterized^29^,^54^ as follows,


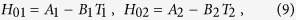


where *B*_*i*_ includes all the local feedbacks: the temperature feedback, the water vapour feedback, the surface albedo feedback, as well as the cloud feedback. Moreover, *B*_*i*_ can be different in different regions. The local feedbacks are usually negative, namely, 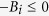, because of the dominant negative temperature feedback (including both surface temperature and lapse rate)^55^,^56^. However, the feedback will not be restricted to be negative everywhere. Physically, a local feedback could become positive for extreme scenarios, because of the strong positive feedbacks from water vapour in the deep tropics^57^ and cloud radiative forcing (CRF), for example, due to the stratus clouds in the subtropics^58^,^59^. The climate system can remain stable in the presence of a weak local positive feedback, e.g., 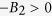, because the atmosphere can transport the extra energy to the other box where the heat is lost to the space efficiently through negative feedback, preventing a local runaway climate instability^57^.

The meridional AHT is assumed linearly proportional to the meridional temperature gradient 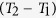
60,61. This Budydo-type model is widely used in the energy balance model^41^,^42^,^43^,^60^ and is more straightforward for interpreting the results, for the purpose of developing a basic understanding of compensating changes in the heat transports.


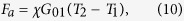


where 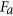 (units: PW, 1 PW = 10^15^ W) is integrated over the 35°N latitude circle.

For the steady state, the total energy in the coupled system is conserved. So, we have,


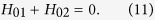


Eq. (11) depicts that the energy gain in the tropical atmosphere-ocean system is equal to the energy loss in the extratropics. Therefore, the total MHT in the climate system is,


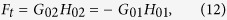


Then, the OHT 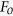 can be obtained as the difference of (12) and (10):


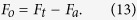


Let us assume there be a perturbation in the system. Eqs. (9) and (11) suggest a relationship of equilibrium temperature changes between two surface boxes,


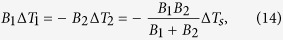


where 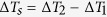. The corresponding changes in heat transport components can then be obtained,


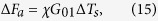



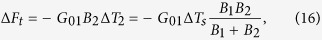



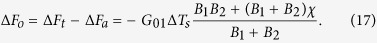


The BJC ratio 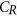 is thus derived as (Supplementary)


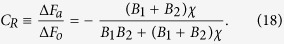


Eq. (18) states that, although the heat transports themselves may have big changes due to temperature change, the ratio between their changes is fixed. In other words, the ratio depends only on two internal parameters: the local climate feedback parameters 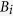, and the atmosphere heat transport efficiency 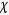
29.

To mimic the climate change over the past 22 kyr, the coupled box model is perturbed by the FWF from the CCSM3 TraCE-21 K. The FWF (units: Sv) used here is the total meltwater flux integrated over the North Atlantic between 35°N and 70°N.

## Additional Information

**How to cite this article**: Yang, H. *et al.* Heat Transport Compensation in Atmosphere and Ocean over the Past 22,000 Years. *Sci. Rep.* **5**, 16661; doi: 10.1038/srep16661 (2015).

## Supplementary Material

Supplementary File
