# Supplementary material for: Heat Transport Compensation in Atmosphere and Ocean over the Past 22,000 Years
Source: Sci Rep. 2015 Nov 16;5:16661. doi: 10.1038/srep16661 (PMC4645171; doi:10.1038/srep16661)
Supplement: Supplementary File [file srep16661-s1.pdf]

Supplementary Information

Heat Transport Compensation in Atmosphere and Ocean over the Past 22,000 Years

Haijun Yang[1,2]*, Yingying Zhao[1], Zhengyu Liu[1,3], Qing Li[1,4], Feng He[3], and Qiong Zhang[5]

[1]*Laboratory for Climate and Ocean-Atmosphere Studies (LaCOAS) and Department of Atmospheric and Oceanic Sciences, School of Physics, Peking University, Beijing 100871, China*

[2]*Qingdao Collaborative Innovation Center of Marine Science and Technology, Qingdao 266003, China*

[3]*Center for Climatic Research, University of Wisconsin, Madison, Wisconsin 53706, USA*

[4]*Department of Earth, Environmental and Planetary Sciences, Brown University, Rhode Island 02912, USA*

[5]*Department of Physical Geography and Bolin Centre for Climate Research, Stockholm University, Stockholm 10691, Sweden*

Contents:

1. Compensating changes over the past 22 kyr
2. Box model
3. Parameters derived from the CCSM3 22-kyr simulation
4. Compensation rate

**Corresponding author address*: Haijun Yang, Department of Atmospheric and Oceanic Sciences, School of Physics, Peking University, 209 Chengfu Road, Beijing China, 100871.

Email: hjyang@pku.edu.cn.

1. Compensating changes over the past 22 kyr

The compensating changes in AHT and OHT are obvious in Fig. S1. Different from Fig. 2, the change in Fig. S1 is defined as the difference between the value in the later period and that in the immediately preceding period. For example, the heat transport change occurred in the OD period is the difference between the heat transport in the OD period and the mean value during the LGM (22-20 ka). The heat transport change occurred in the BA is obtained by subtracting the mean value during the OD (17-15 ka). The heat transport change in the YD is obtained by subtracting the mean value during the BA (14.5-12.8 ka). The heat transport change in the Holocene is obtained by subtracting the mean value during the YD (12.7-11.5 ka). The long-term linear trends over the past 22 kyr are not removed before calculating these differences. This approach can illustrate the compensating changes between the AHT and OHT more clearly, in accompany with several big climate shifts since the LGM.

The changes in AHT and OHT compensate each other very well at most latitudes in response to all climate shifts except that from the YD to the Holocene in the northern high latitudes (Fig. S1b). The correlations between AHT changes and OHT changes are generally high (exceeding 0.90) in most regions.

2. Coupled box model

2.1. Basic equations

The coupled model consists of two atmosphere boxes and four ocean boxes, as illustrated in Fig. S2. The two-box atmosphere covers one hemisphere and the four-box ocean spans an arbitrary longitude range (60° for the Atlantic sector or 180° for the global Northern Hemisphere ocean) between the equator and 75°N. The latitude of 35°N divides the atmosphere and ocean box, where the zonal mean net radiative forcing is close to zero and is positive (negative) to the south (north). The northward transports of heat and water vapour in the atmosphere are near their peaks at 35°N. The box ocean model originally appeared in Stommel (1961) and was developed further in many studies (Marotzke, 1990; Huang et al., 1992; Nakamura et al., 1994 (NSM94); Tziperman et al., 1994; Marotzke and Stone, 1995 (MS95)).

The ocean boxes are governed by eight equations (c.f., Stommel 1961, Marotzke, 1990; Huang et al., 1992; NSM94; Tziperman et al., 1994; MS95; Yang et al., 2015):

$$\dot{T}_1 = \frac{1}{\epsilon c \rho_0 D_1}[(A_1 - B_1 T_1) + \chi(T_2 - T_1)] + q(T_2 - T_1), \quad (1a)$$

$$\dot{T}_2 = \frac{1}{\epsilon c \rho_0 D_1}[(A_2 - B_2 T_2) - \chi(T_2 - T_1)] + q(T_4 - T_2), \quad (1b)$$

$$\dot{T}_3 = q(T_1 - T_3), \quad (1c)$$

$$\dot{T}_4 = q(T_3 - T_4). \quad (1d)$$

$$\dot{S}_1 = -\frac{S_0}{\epsilon_w D_1}\gamma(T_2 - T_1) + q(S_2 - S_1), \quad (2a)$$

$$\dot{S}_2 = +\frac{S_0}{\epsilon_w D_1}\gamma(T_2 - T_1) + q(S_4 - S_2), \quad (2b)$$

$$\dot{S}_3 = q(S_1 - S_3), \quad (2c)$$

$$\dot{S}_4 = q(S_3 - S_4). \quad (2d)$$

where A_1 (<0) and A_2 (>0) are net incoming radiation (Wm^{-2}) at high and low latitudes, respectively; B_1 and B_2 are climate feedback parameters at high and low latitudes, respectively; χ and γ are the efficiencies of atmosphere heat and moisture transports; c is the seawater specific heat capacity; ρ_0 is the seawater density; and S_0 is a constant reference salinity (35 psu). $\epsilon \equiv G_1/G_{01} = G_2/G_{02}$, which indicates relative ocean coverage in both high and low latitude areas; here, G_{01} and G_{02} are the entire areas of the two atmosphere boxes separated by 35°N; G_1 and G_2 are the areas of corresponding ocean boxes. For simplicity, we assume $G_1 = G_2$ and $G_{01} = G_{02}$. If an aquaplanet is studied, $\epsilon = 1$; otherwise, $\epsilon < 1$. $\epsilon_w \equiv G_1'/G_{01}$, where G_1' depicts the ocean area as well as the catchment area of the ocean basin, which includes the influence of river runoff in the oceanic freshwater budget. It is obvious that $G_1' > G_1$. All parameters used in this study are listed in Table 1, which are based on NSM94, MS95 and our coupled model simulations (Yang et al., 2014) as well as observations.

The flow strength, or the volume transport due to the THC between two adjacent ocean boxes, q, is assumed to be linearly proportional to the density difference between two surface ocean boxes 1 and 2 (NSM94) and given by

$$q = \kappa[\alpha(T_2 - T_1) - \beta(S_2 - S_1)] = \kappa(\alpha T_s - \beta S_s), \quad (3)$$

where κ is a constant parameter that sets the reference flushing (turnover) timescale for a surface ocean box (units: s^{-1}); α and β are the thermal and haline expansion coefficients of seawater, respectively. This simple relationship for q is supported by many OGCM studies (e.g., Hughes and Weaver, 1994). In this study, we focus on a positive (northward) meridional mass transport ($q > 0$), which assumes a temperature-dominated circulation ($\alpha T_s > \beta S_s$) and is driven by the cooling and sinking in the extratropical (northern) box and heating in the tropical box.

The meridional atmosphere and ocean heat transports are parameterized as follows:

$$F_a = \chi G_{01}(T_2 - T_1)\ , \quad (4)$$

$$F_o = c\rho_0 \epsilon G_{01} D_1 q(T_2 - T_3)\ . \quad (5)$$

In Eq. (1), a simple parameterization of the net radiative forcing (Wm^{-2}) at the TOA is used, which is widely employed in EBMs (e.g., North, 1975; Wang and Stone, 1980; NSM94),

$$H_{01} = A_1 - B_1 T_1\ , \quad H_{02} = A_2 - B_2 T_2\ . \quad (6)$$

The surface heat fluxes combining the AHT and the net radiation fluxes at the TOA are,

$$H_1 = \frac{1}{\epsilon c \rho_0 D_1}[(A_1 - B_1 T_1) + \chi(T_2 - T_1)]\ , \quad (7a)$$

$$H_2 = \frac{1}{\epsilon c \rho_0 D_1}[(A_2 - B_2 T_2) - \chi(T_2 - T_1)]\ . \quad (7b)$$

The spatially averaged ocean heat uptake is,

$$H_1 + H_2 = \frac{1}{\epsilon c \rho_0 D_1}(H_{01} + H_{02}) = \frac{1}{\epsilon c \rho_0 D_1}(A_1 + A_2 - B_1 T_1 - B_2 T_2), \quad (8)$$

which is independent of the AHT. The oceanic heat budget, as a whole, is only determined by the net radiative forcing at the TOA:

$$\dot{T}_1 + \dot{T}_2 + \dot{T}_3 + \dot{T}_4 = H_1 + H_2 = \frac{1}{\epsilon c \rho_0 D_1}(H_{01} + H_{02}). \quad (9)$$

At the steady state, the total energy in the six-box system is conserved. Eq. (9) becomes,

$$H_1 + H_2 = H_{01} + H_{02} = 0\ , \quad (10)$$

which depicts that the ocean heat uptake in the tropical box is equal to the ocean heat release in the extratropical box; in other words, the energy gain in the tropical atmosphere-ocean system is equal to the energy loss in the extratropics.

In Eqs. (1)-(2), we also follow the simplest assumptions that the meridional heat and moisture transports are linearly proportional to the meridional temperature gradient $(T_2 - T_1)$ (Budydo, 1969). This Budydo-type model is widely used in the EBMs (e.g., Lindzen and Farrell, 1977; North, 1984; Stone and Yao, 1990) and is more straightforward for interpreting the results, which helps to develop a basic understanding of compensating changes in heat transports.

Without external freshwater sources, the total salt of the model ocean is conserved:

$$\dot{S}_1 + \dot{S}_2 + \dot{S}_3 + \dot{S}_4 = 0 \tag{11}$$

2.2. Equilibrium solutions

The equilibrium states of temperature and salinity as well as AHT and OHT can be obtained by simply letting temporal tendency be zero ($\dot{T}_i = 0,\ \dot{S}_i = 0$):

$$B_1 T_1 + B_2 T_2 = A_1 + A_2, \tag{12a}$$

$$T_1 = T_3 = T_4, \tag{12b}$$

$$S_1 = S_3 = S_4. \tag{12c}$$

$$F_o = G_{01}(H_{02} - \chi T_s), \tag{13a}$$

$$F_a = \chi G_{01} T_s, \tag{13b}$$

$$F_t = F_o + F_a = G_{01} H_{02} = -G_{01} H_{01}. \tag{13c}$$

For reasonable values of A_1, A_2 and B_1, B_2 (Table 1), the surface mean temperature is about 15°C. Using the reference parameters in Table 1, the mean climate can be determined. The model mean climate is tuned to be consistent with those of observations, NSM94 and MS95, and particularly the CCSM3 TraCE-21K simulation.

2.3. Bjerknes compensation

Assuming that there is a perturbation in the system, Eq. (12a) suggests a relationship of equilibrium temperature changes between the two surface ocean boxes,

$$B_1 \Delta T_1 = -B_2 \Delta T_2 = -\frac{B_1 B_2}{B_1 + B_2} \Delta T_s \,, \qquad (14)$$

where $\Delta T_s = \Delta T_2 - \Delta T_1$. The corresponding changes in heat transport components can be obtained from (13),

$$\Delta F_o = -G_{01}(B_2 \Delta T_2 + \chi \Delta T_s) = -G_{01} \Delta T_s \frac{B_1 B_2 + (B_1 + B_2)\chi}{B_1 + B_2} \,, \qquad (15a)$$

$$\Delta F_a = \chi G_{01} \Delta T_s \,, \qquad (15b)$$

$$\Delta F_t = -G_{01} \Delta T_s \frac{B_1 B_2}{B_1 + B_2} \,. \qquad (15c)$$

The BJC ratio C_R is thus defined, as in Swaluw et al. (2007) and Rose and Ferreira (2013):

$$C_R \equiv \frac{\Delta F_a}{\Delta F_o} = -\frac{(B_1 + B_2)\chi}{B_1 B_2 + (B_1 + B_2)\chi} \,. \qquad (16)$$

Eq. (16) states that C_R is independent of the mean climate, changes in T_s and S_s, as well as the heat transports themselves. It is determined by only two climate parameters: the local climate feedback parameter B_i, and the AHT coefficient χ.

C_R is always negative for a stable climate system with total energy conservation. The local climate feedback B_i must be in a reasonable range to maintain the stability of the six-box model. This requires a global mean negative feedback first:

$$-(B_1 + B_2) < 0 \, , \tag{17}$$

and a satisfaction of the stability condition:

$$-(B_1 + B_2) < B_1 B_2/\chi \, . \tag{18}$$

Eq. (16) suggests that the changes in AHT and OHT can be undercompensated, perfectly compensated, or overcompensated, depending on how to treat the heat efficiency χ/B, or more specifically, the local climate feedback B, because χ is always positive and less uncertain, while B is spatial-temporal dependent and could include various positive or negative feedback processes. Stronger negative local climate feedback B will result in smaller C_R. Strong climate feedback (both positive and negative) means small surface temperature perturbations can cause big changes in the TOA energy flux, which suggests a serious violation of the fundamental physical assumption made in the BJC. Then, C_R will deviate significantly from 1. Mathematically, this is depicted as,

$$|C_R| \sim \begin{cases} < 1, & if \;\; B_1 B_2 > 0 \quad Undercompensation \\ = 1, & if \;\; B_1 B_2 = 0 \quad Full\ compensation \\ > 1, & if \;\; B_1 B_2 < 0 \quad Overcompensation \end{cases} . \tag{20}$$

Finally, for the convenience of later discussion the equilibrium temperature changes can be re-written in terms of OHT change ΔF_o as follows,

$$\Delta T_1 = \frac{B_2 \Delta F_o / G_{01}}{B_1 B_2 + (B_1 + B_2)\chi}, \quad \Delta T_2 = \frac{-B_1 \Delta F_o / G_{01}}{B_1 B_2 + (B_1 + B_2)\chi} \, . \tag{21}$$

Table 1 Parameters used in this study.

Symbol	Physical meaning	Units	Value	Reference	Note
A_1<br>A_2	Net incoming radiative at box 1 and 2	W m^{-2}	-40<br>90	MS95	
B_1<br>B_2	Local climate feedback parameter at box 1 and box 2	W m^{-2} K^{-1}	-0.4<br>1.7		
$c\rho_0$	Heat capacity of a unit water volume	J m^{-3} K^{-1}	4×10^{6}	MS95	
D_1<br>D_2	Depth of upper and lower boxes	m	400<br>4000		
G_{01}	Entire surface area north of the dividing latitude	m^2	1.25×10^{14}	MS95	
L_1<br>L_2	Meridional scale of low and high latitude boxes	degree	35°<br>40°	MS95	
S_0	Reference salinity	psu	35.0	MS95	
α	Thermal expansion coefficient	K^{-1}	2.5×10^{-4}	MS95	
β	Haline contraction coefficient	psu^{-1}	7.5×10^{-4}	MS95	
ϵ	Ratio of ocean area of box 1 to F_{01}, i.e., G_1/G_{01}, $\epsilon \leq 1$	-	0.15~0.2	MS95	Atlantic sector
ϵ_w	Ratio of ocean and catchment area to G_{01}, $\epsilon \leq \epsilon_w \leq 1$	-	0.3	MS95	
κ	Advective timescale coefficient	s^{-1}	1.9×10^{-7}		D=5km
γ	Atmospheric moisture transport coefficient divided by G_{01}	m s^{-1} K^{-1}	2.3×10^{-10}		
χ	Atmospheric heat transport coefficient divided by F_{01}	W m^{-2} K^{-1}	1.5		

3. Parameters derived from the CCSM3 TraCE-21K simulation

3.1. Atmospheric heat transport coefficient χ

Based on Eq. (4), χ can be determined using the output from the TraCE-21K. Figure S3 shows the AHT at 35°N and T_s between the tropical and extratropical boxes. During the past 22 kyr, the AHT at 35°N ranged between 5.2 and 5.8 PW, within ±6% of the mean value (5.5 PW). For different periods, the AHT varied slightly and nearly linearly with T_s. During the OD, the bigger T_s corresponded to a stronger AHT, which was related to the weakened THC. During the Holocene, the weaker AHT resulted from a weaker T_s, corresponding to the stronger THC that was close to the present climate. χ in the LGM and OD was around 1.4 W m^{-2} K^{-1}, and it was about 1.6 W m^{-2} K^{-1} in the Holocene (Fig. S3, inset). In general, χ did not change much over the past 22 kyr; its mean value was 1.5 W m^{-2} K^{-1}.

3.2. Climate feedback parameters B_1 *and* B_2

Climate feedback and sensitivity are extremely complicated, and have been studied extensively by many researchers (e.g., Bates, 1999, 2007, 2010; Hwang and Frierson, 2010; Hwang et al., 2011). Local climate feedback strength determines atmosphere behaviours to a great extent, such as how the AHT would respond to surface temperature change. The relationship between local feedback strength and AHT was studied by Hwang and Frierson (2010), Hwang et al. (2011), Zelinka and Hartmann (2011), Feldl and Roe (2013) and Huang and Zhang (2014). Rose et al. (2014) explicitly showed how the AHT responded to prescribed patterns of surface heat flux (analogous to the OHT in equilibrium) in different feedback regimes, using both simple EBM and GCM.

In this study, we do not intend to investigate thoroughly how local climate feedbacks can be identified correctly; instead, we hope to understand the general relationship between the net heat flux

at the TOA and surface temperature using a conceptual model. We are interested in the sign of the feedback (positive or negative) and its approximate strength at a given latitude.

Simple linear regression is used to determine the feedback parameters (B_1, B_2) between the heat flux at the TOA and surface temperature based on Eq. (6). Similar approach was used in Rose and Marshall (2009). The data are from the CCSM TraCE-21K simulation (He, 2011). Before calculating the linear regression, all data are averaged over the tropical (0°-35°N, 0°-360°) and extratropical (35°-75°N, 0°-360°) boxes. The surface temperature includes the SST in the ocean and SAT over the land. The linear regression is applied to the net heat flux, the net downward shortwave (SW) flux and the net outgoing longwave (LW) flux, all at the TOA. The regression coefficients represent the feedback parameters B_1 and B_2.

Based on the TraCE-21K, the extratropical region shows a weak positive feedback ($-B_1 > 0$) between the net heat flux and surface temperature (Fig. S4a). This positive feedback is mainly due to the strong positive feedback between SW and surface temperature (Fig. S4e), closely related to low cloud changes (Zhang et al., 2010), which overcomes the strong negative feedback between LW and surface temperature (Fig. S4c). The tropical region shows a negative feedback ($-B_2 < 0$) between the net heat flux and surface temperature (Fig. S4b). This negative feedback is mainly due to the strong negative feedback between LW and surface temperature (in association with high clouds) (Fig. S4d), which dominates the positive feedback between SW and surface temperature (Fig. S4f).

We can see that climate feedback parameters varied with time (Fig. S4). Different colors show different periods. Generally, the LW feedback was stable in both tropics and extratropics (Figs. S4c-d). The SW feedback was stable in the extratropics (Fig. S4e) but varied dramatically in different periods in the tropics (Fig. S4f). For example, during the LGM (green in Fig. S4f), very strong SW positive feedback occurred in the tropics, which eventually resulted in strong positive feedback in the

total net heat flux (green in Fig. S4b). Combined with the weak positive feedback in the extratropics (Fig. S4a), one may conclude that the LGM climate was overall unstable (though this argument needs more thorough study). The feedback parameters (B_1, B_2) are listed in Table 2.

Table 2 Climate feedback parameters.

Symbol		LGM	OD	BA	YD	HC	Mean
χ		1.42	1.40	1.54	1.53	1.59	**1.50**
B_1	LW	1.39	1.25	1.49	1.20	1.25	1.31
	SW	-1.87	-1.72	-1.97	-1.53	-1.67	-1.75
	NET	-0.48	-0.47	-0.48	-0.33	-0.43	**-0.44**
B_2	LW	1.39	1.07	2.55	1.71	2.29	1.80
	SW	-6.39	-0.12	-0.94	-1.21	-1.13	-1.96
	NET	-13.37	0.95	1.61	1.79	2.65	**1.75***
C_R	LW	-0.67	-0.71	-0.62	-0.68	-0.66	-0.66
	SW	54.6	-1.09	-1.70	-1.79	-1.73	-2.61
	NET	-1.49	-2.95	-1.79	-1.36	-1.47	**-1.64**

*Mean from the OD to the HC.

4. Compensation rate

Finally, the BJC rate can be estimated using the TraCE-21K output. Figure S5 shows C_R with respect to B_1 and B_2 based on Eq. (16). C_R derived from the CCSM3 TraCE-21K simulation (listed in Table2) are marked by color symbols. The overall feedbacks between the net heat flux at the TOA and surface temperature are marked by green color, showing a weak positive (strong negative) feedback in the extratropics (tropics) for OD, BA, YD, and HC.

References

Armour, et al., 2013: Time-varying climate sensitivity from regional feedbacks. *J. Clim.*, **26**, 4518-4534.

Bates, 1999: A dynamical stabilizer in the climate system: a mechanism suggested by a simple model. *Tellus*, **51A**, 349-372.

Bates, 2007: Some considerations of the concept of climate feedback. *Q. J. R. Meteorol. Soc.*, **133**, 545-560.

Bates, 2010: Climate stability and sensitivity in some simple conceptual models. *Clim. Dyn*., DOI 10.1007/s00382-010-0966-0.

Bjerknes, J., 1964: Atlantic air/sea interaction. *Advances in Geophysics*, Vol. **10**, Academic Press, 1–82.

Clement, A. C., R. Burgman, and J. R. Norris, 2009: Observational and model evidence for positive low-level cloud feedback. *Science*, **325**, 460-464.

Feldl and Roe, 2013: The nonlinear and nonlocal nature of climate feedbacks. *J. Clim.*, **26**, 8289-8304.

He, F., 2011: Simulating transient climate evolution of the Last Deglaciation with CCSM3. Ph.D thesis, University of Wisconsin-Madison, 1-177.

Huang, R. X., J. R. Luyten, and H. M. Stommel, 1992: Multiple equilibrium states in combined thermal and saline circulation. *J. Phys. Oceanogr*., **22**, 231-246.

Huang and Zhang, 2014: The implication of radiative forcing and feedback for meridional energy transport. *Geophys. Res. Lett.*, **41**, 1665-1672.

Hughes, T. C. M., and A. J., Weaver, 1994: Multiple equilibria of an asymmetric two-basin model. *J. Phys. Oceanogr*., **24**, 619-637.

Hwang and Frierson, 2010: Increasing atmospheric poleward energy transport with global warming. *Geophys. Res. Lett.*, **7**, L24807.

Hwang et al., 2011: Coupling between Arctic feedbacks and changes in poleward energy transport. *Geophys. Res. Lett.*, **38**, L17704.

Marotzke J., 1990: Instabilities and multiple equilibria of the thermohaline circulation. Ph.D. thesis. Ber. Inst. Meeresk. Kiel, Germany, 126 pp.

Marotzke J., and P. Stone, 1995: Atmospheric transports, the thermohaline circulation, and flux adjustments in a simple coupled model. *J. Phys. Oceanogr*., **25**, 1350-1364.

Nakamura, M., P. H. Stone, and J. Marotzke, 1994: Destabilization of the thermohaline circulation by atmospheric eddy transports. *J. Clim.*, **7**, 1870-1882.

Pierrehumbert , R., 1995: Thermostates, Radiator fins and the local runaway greenhouse. *J. Atmos. Sci*., **52,** 1784-1805.

Philander, S. G. H., D. Gu, D. Halpern, G. Lambert, N.-C. Lau, T. Li, and R. C. Pancanowiski, 1996: Why the ITCZ is mostly north of the Equator. *J. Clim.*, **9**, 2958-2972.

Rose, B. E., and J. Marshall, 2009: Ocean heat transport, sea ice, and multiple climate states: insights from energy balance models. *J. Atmos. Sci.*, **66**, 2828-2843.

Rose, B. E., and D. Ferreira, 2013: Ocean heat transport and water vapor greenhouse in a warm equable climate: a new look at the low gradient paradox. J. Clim., 26, 2117-2136.

Rose et al., 2014: The dependence of transient climate sensitivity and radiative feed- backs on the spatial pattern of ocean heat uptake. *Geophys. Res. Lett.*, **41**, doi:10.1002/2013GL058955.

Stommel, H., 1961: Thermohaline convection with two stable regimes of flow. *Tellus*, **13**, 224-230.

Stone, P. H., 1978: Constraints on dynamical transports of energy on a spherical planet. *Dynamics of Atmospheres and Oceans*, **2**, 123–139.

Stone, P. H., and M.–S. Yao, 1990: Development of a two-dimensional zonally averaged statistical-dynamical model. Part III: The parameterization of the eddy fluxes of heat and moisture. *J. Clim.*, **3**, 726-740.

Soden, B. J., A. J. Broccoli, and R. S. Hemler, 2004: On the use of cloud forcing to estimate cloud feedback. *J. Climate*, **17**, 3661–3665.

Tziperman, E., J. R. Toggweiler, Y. Feliks, and K. Bryan, 1994: Instability of the thermohaline circulation with respect to mixed boundary conditions: Is it really a problem for realistic models. *J. Phys. Oceanogr.*, **24**, 217-232.

Wang, W.-C., and P. H. Stone, 1980: Effect of ice-albedo feedback on global sensitivity in a one-dimensional radiative-convective climate model. *J. Atmos. Sci.*, **37**, 545-552.

Watterson, 2003: Effects of a dynamic ocean on simulated climate sensitivity to greenhouse gases. *Clim. Dyn.*, doi: 10.1007/s00382-003-0326-4.

Yang, H., Q. Li, K. Wang, Y. Sun, and D. Sun, 2014: Decomposing the meridional heat transport in the climate system. *Clim. Dyn.*, doi: 10.1007/s00382-014-2380-5.

Yang, H., Y. Zhao, and Z. Liu, 2015: Understanding Bjerknes compensation in atmosphere and ocean heat transports using a coupled box model. *J. Clim.*, submitted.

Zelinka and Hartmann, 2011: Climate Feedbacks and Their Implications for Poleward Energy Flux Changes in a Warming Climate. *J. Clim.*, **25**, 608-624.

Zhang, M., J. Hack, J. Kiehl, and R. Cess, 1994: Diagnostic study of climate feedback processes in atmospheric general circulation models. *J. Geophys. Res*., **99**, 5525-5537.

Zhang R., S. M. Kang, and I. M. Held, 2010: Sensitivity of climate change induced by weakening of the Atlantic Meridional Overturning Circulation to cloud feedback. *J. Clim*., **23**, 378-389.

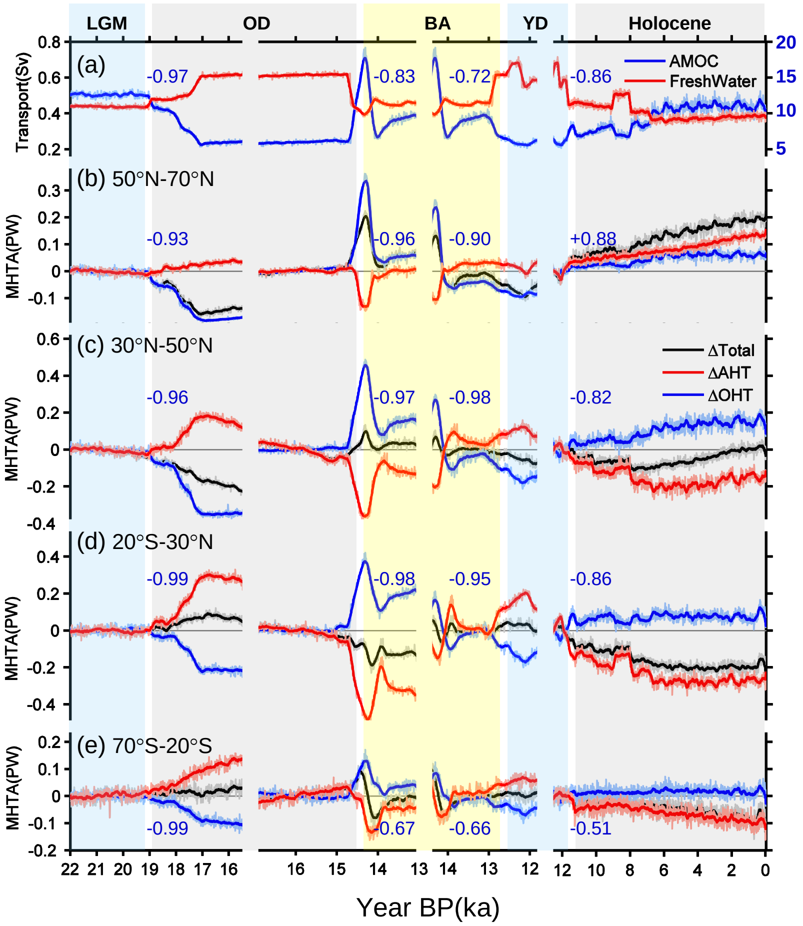

Figure S1 AMOC, FWF and MHT. a, The blue curve shows the AMOC index (Sv), and the red curve shows the FWF (Sv) obtained by integrating the total melting water flux over the North Atlantic between 35° and 70°N. The number in parentheses indicates the correlation coefficient between the AMOC and FWF. **b-e**, Anomalous MHT averaged over different latitude bands (PW). Anomalous heat transports are obtained by subtracting the mean values in the immediately preceding period from the heat transport in the current period (see Methods for data sources, etc.). In **b-e**, the black curve is the total MHT; red, the AHT; and blue, the OHT. The numbers in blue indicate correlation coefficients between AHT and OHT. The thick solid curves in each panel are the low-pass-filtered (150-year running mean) versions of the time series.

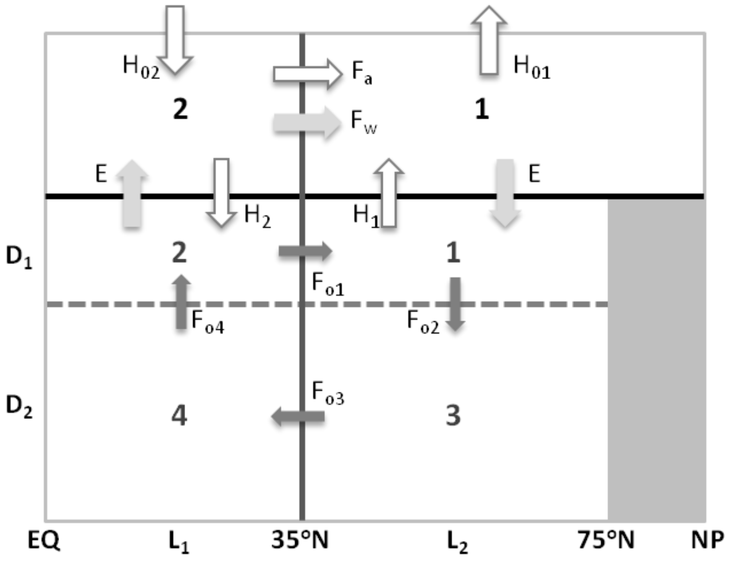

Figure S2 Conceptual 6-box coupled model. Boxes 1 and 3 represent the upper and lower layers of the extratropical ocean, respectively, and boxes 2 and 4, of the tropical ocean. D_1 and D_2 are the depths of upper and lower layers, respectively. L_1 and L_2 are the meridional scales of tropical and extratropical boxes, respectively. H_1 and H_2 are ocean heat gains through the sea surface in the extratropical and tropical oceans, respectively. E is the net freshwater loss at the low latitudes, or the net freshwater gain at the high latitudes. H_{01} and H_{02} are the net energy gains at the top of the atmosphere (TOA) in the extratropics and tropics, respectively. F_a is the meridional atmosphere energy transport. F_w is the meridional atmosphere moisture transport. $F_{o1\text{-}4}$ illustrates qualitatively the heat transport among different boxes.

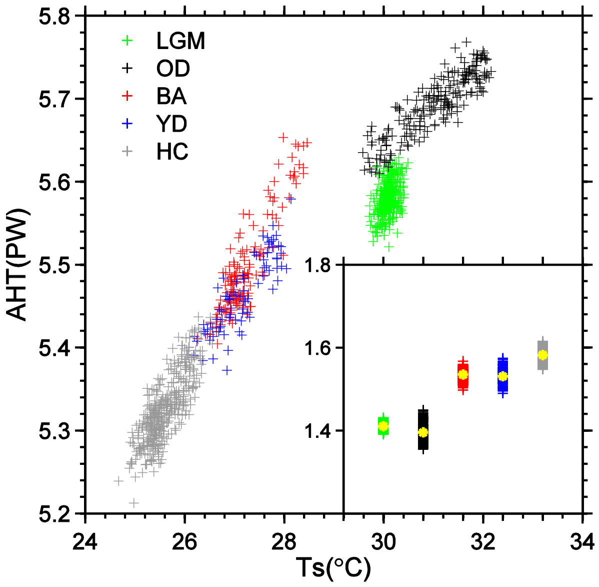

Figure S3 AHT vs. T_s, and χ. Scattering plot shows relationship between the AHT at 35°N and T_s based on the CCSM3 TraCE-21K simulation. Different colors represent different periods. Inset shows the values of atmospheric heat transport coefficient χ for different periods, based on Eq. (4). The TraCE-21K simulation gives an average χ =1.5 W m^{-2} K^{-1}.

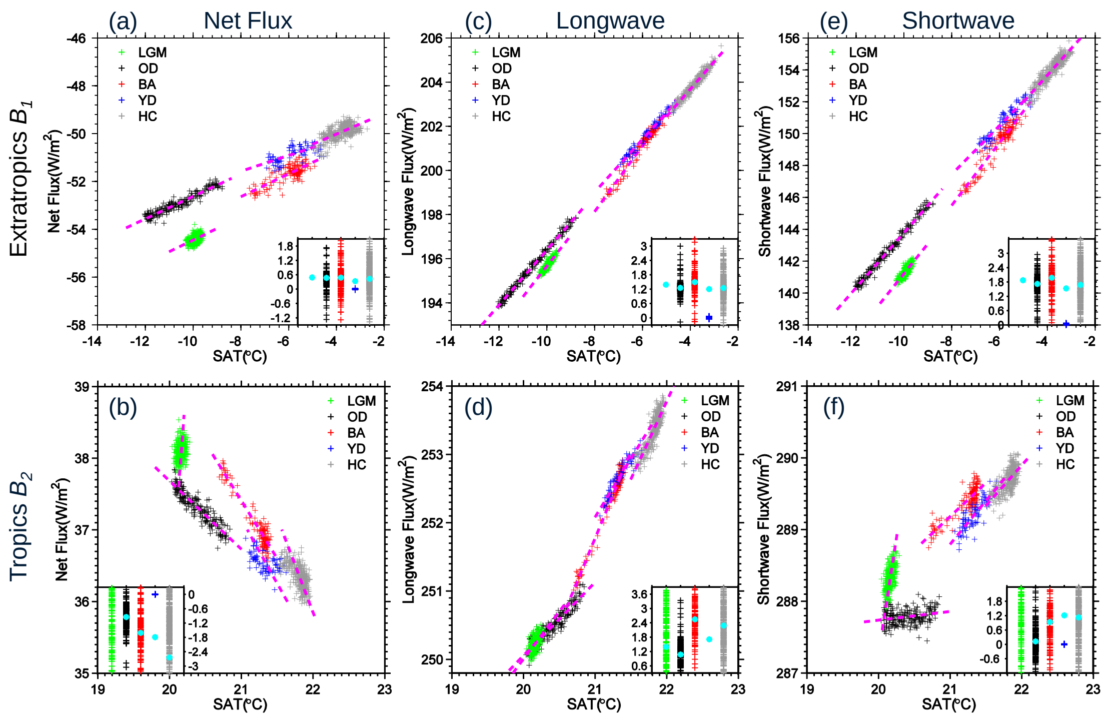

Figure S4 Linear regression of heat flux at the TOA with respect to surface temperature. **a-b**, Dashed lines show linear regressions of the net heat flux at the TOA with respect to surface temperature. Data are from the CCSM3 TraCE-21K simulation and averaged over the extratropical box (35°-70°N) and tropical box (0°-35°N). Inset shows climate feedback B_1 and B_2 obtained from the linear regression. On average, $-B_1$=0.4 and $-B_2$=-1.7, showing a weak positive feedback in the extratropics and a strong negative feedback in the tropics between the net heat flux at the TOA and surface temperature. **c-d,** Same as **a-b**, except for the outgoing longwave (LW) flux. On average, $-B_1$=-1.3 and $-B_2$=-1.8, showing strong negative feedbacks between LW and surface temperature in both regions. **e-f,** Same as **a-b**, except for the incoming shortwave (SW) flux. On average, $-B_1$=1.7 and $-B_2$=1.9, showing positive feedbacks between SW and surface temperature in both regions. Different colors indicate different periods.

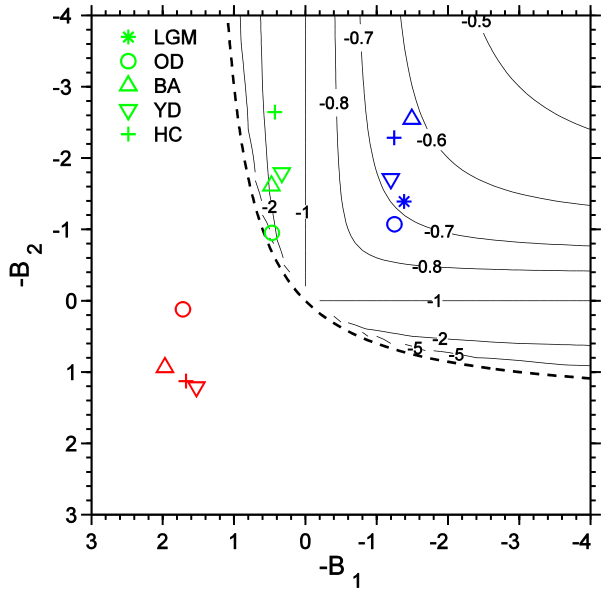

Figure S5 BJC rate (C_R) with respect to B_1 and B_2. Solid curves are C_R based on Eq. (16) with χ=1.5 W m^{-2} K^{-1}; dashed line indicates stability condition. Green symbols are C_R with climate feedback parameters (B_1, B_2) for net heat flux at the TOA; blue symbols, C_R with B_1, B_2 for net outgoing LW flux at the TOA; red symbols, C_R with B_1, B_2 for net incoming SW flux at the TOA. B_1, B_2 are obtained using **Figure S4**. Different symbols represent different periods of the CCSM3 TraCE-21K simulation.
